# Upfront Xpert MTB/RIF testing on various specimen types for presumptive infant TB cases for early and appropriate treatment initiation

**DOI:** 10.1371/journal.pone.0202085

**Published:** 2018-08-30

**Authors:** Neeraj Raizada, Sunil D. Khaparde, Raghuram Rao, Aakshi Kalra, Sanjay Sarin, Virender Singh Salhotra, Soumya Swaminathan, Ashwani Khanna, Kamal Kishore Chopra, M. Hanif, Varinder Singh, K. R. Umadevi, Sreenivas Achuthan Nair, Sophie Huddart, Rajneesh Tripathi, C. H. Surya Prakash, B. K. Saha, Claudia M. Denkinger, Catharina Boehme

**Affiliations:** 1 Foundation for Innovative New Diagnostics, New Delhi, India; 2 Central TB Division, Government of India, New Delhi, India; 3 Indian Council of Medical Research, New Delhi, India; 4 State TB office, Govt of NCT, New Delhi, India; 5 New Delhi TB Centre, New Delhi, India; 6 Lady Hardinge Medical College and assoc Kalawati Saran Children's Hospital, New Delhi, India; 7 National Institute of research in Tuberculosis, Chennai, India; 8 World Health Organization, Country Office for India, New Delhi, India; 9 McGill University, Montreal, Canada; 10 Intermediate Reference Laboratory, Hyderabad, India; 11 Intermediate Reference Laboratory, Kolkata, India; 12 Foundation for Innovative New Diagnostics, Geneva, Switzerland; The Ohio State University, UNITED STATES

## Abstract

**Background:**

Diagnosis of tuberculosis (TB) in infants is challenging due to non-specific clinical presentations of the disease in this age-group and low sensitivity of widely available TB diagnostic tools, which in turn delays prompt access to TB treatment. Upfront access to Xpert/MTB RIF (Xpert) testing, a highly sensitive and specific rapid diagnostic tool, could potentially address some of these challenges. Under the current project, we assessed the utility and feasibility of applying upfront Xpert for diagnosis of tuberculosis in infants, including for testing of non-sputum specimens.

**Methods:**

A high throughput lab was established in each of the four project cities, and linked to various health care providers across the city, through rapid specimen transportation and electronic reporting linkages. Free Xpert testing was offered to all infant (<2 years of age) presumptive TB cases (both pulmonary and extra-pulmonary) seeking care at public and private health facilities.

**Results:**

A total of 7,994 presumptive infant TB cases were enrolled in the project from April 2014 to October 2016, detecting 465 (5.8%, CI: 5.3–6.4) TB cases. The majority (93.9%; CI: 93.4–94.4) of patient specimens were non-sputum and TB positivity was higher amongst non-sputum specimens. Further, a high proportion (5.6% CI 3.8–8.1) of infant TB cases were found to be rifampicin resistant. Covering large cities with a single lab per city over more than two years, the project demonstrated the feasibility of same-day diagnosis with upfront Xpert testing. This in turn led to prompt treatment initiation, with a two-day median turnaround time to treatment initiation. Case mortality observed in the project cohort of diagnosed TB cases was 11.0% (CI 8.4–14.1), the majority of which was pre- or early treatment mortality, in spite of prompt access to treatment for most diagnosed cases.

**Conclusion:**

The current project demonstrated the feasibility of applying rapid and upfront Xpert testing for presumptive infant TB cases. Rapid TB diagnosis in turn facilitates prompt and appropriate treatment initiation. Further, levels of rifampicin resistance observed in infants TB cases highlight the additional benefit of upfront resistance testing. However, high rates of early case mortality, in spite of prompt diagnosis and treatment initiation, highlight the need for further research in infant patient pathways for overall improvement in TB care for infant populations.

## Introduction

In high burden tuberculosis (TB) settings, it is estimated that childhood TB contributes to 15–20% of all TB cases and is one of the leading cause of childhood mortality [1, 2]. India is the country with the highest TB and DR-TB (drug resistant TB) burden. Over one quarter of the world’s MDR-TB (Multi-drug resistant TB) infected individuals live in India [3]. In a recently conducted Indian Anti-TB Drug Resistance Survey, it was found that among the 4,958 TB patients with Drug susceptibility testing (DST) results, 28% had resistance to one or more anti TB drugs, while 6.19% had MDR-TB [4]. In 2016, 76,475 pediatric TB cases were notified in India, accounting for about 5% of notified TB cases [5, 6]. However, poor ascertainment and reporting of cases of TB prevent accurate estimation of the burden of TB in infants (<2 years of age) and actual numbers are likely higher [2, 7, 8].

Diagnosis of TB in infants is largely based on the combination of symptoms, positive history of contact with a TB case, clinical and radiological findings and a tuberculin skin test (TST) [7–9]. Microbiological confirmation is often not possible due to difficulties in obtaining a sputum specimen and the poor performance of traditional microbiological tests [9–13]. Low diagnostic yield, using a combination of smear microscopy and culture has been observed in infants known to have highly probable TB based on clinic-epidemiological evidence [14, 15]. Furthermore, long turnaround time for detecting *Mycobacterium tuberculosis* by culture limits its utility in routine TB case management decisions. As a result, microbiologic confirmation of TB is still rarely attempted.

The existing India-based pathways focus on adult patients. Specifics, relevant to pediatric TB diagnosis are not clearly outlined. The few studies which have captured pediatric pathways to care, highlight the often substantial delay in TB diagnosis and identify the first point of contact for care-seeking to be the local provider in the private sector [16, 17].

TB in infants is also often underrecognized and underreported due to the diversity in clinical presentations and course of the disease, which make clinical diagnosis challenging. For TB diagnosis in the pediatric age group, including infants, diagnostic algorithmic approach based on clinical, radiological and TST have shown variable case yield and agreement with gold standards, and its performance varies across different settings [18]. In routine practice, TB in infants is usually diagnosed based on history of contact with a known TB cases, and/or by exclusion after lack of response to prescribed antibiotic treatment. In the absence of history of contact with known TB cases, TB is rarely suspected in infants which further delays access to TB treatment. Upfront access to rapid and accurate diagnosis of TB is necessary to address this critical diagnostic gap in this highly vulnerable population. A review of the literature provides limited insights into the utility of Xpert for TB diagnosis in infants. Most published studies were either individual facility based and/or based on a very small sample size [19]. This underscores the need for further research for better insights and systematic approach to TB care in this highly vulnerable population, both from individual case management and epidemiological perspective.

WHO endorsed Xpert MTB/RIF (Xpert) for TB diagnosis in presumptive pediatric TB cases and extra-pulmonary tuberculosis (EPTB) cases [20–24]. In line with this global guidance, a project was undertaken by FIND, under India’s Revised National TB Control Program (RNTCP), in four major cities.

The project represents by far the largest initiative to date, to our knowledge, globally, dedicated to a pediatric population. Within this project, a large cohort of presumptive infant TB cases was given upfront access to Xpert testing and quality TB treatment was ascertained for all Xpert-positive cases. The project was initially undertaken as a pilot mode from April to November 2014 and provided promising results in children of 0–14 years old [25].

This report now focuses specifically on the intervention for infants (<2 years of age), which has its own specific diagnostic and treatment challenges and is relatively less studied. The article addresses several aspects, which are crucial for scalability and replicability of the intervention.

## Material and methods

The project was implemented in four major Indian cities, Chennai, Delhi, Hyderabad and Kolkata, covering a population of 30 million, with the objective of providing upfront free-of-cost access to Xpert testing for all presumptive pediatric TB cases. We report here the sub-analysis of presumptive infant TB cases (<2 years of age) covered under the project. While the exact estimate of infant population in these cities is not available, the project interventions were offered to all infants identified as presumptive TB cases from April 2014 to October 2016. The project established a high throughput Xpert laboratory in each city that was dedicated to the pediatric population. Using a hub and spoke model, each laboratory was linked to various public and private sector health care providers in the city. Specimen transportation linkages were optimized, taking into account feasible local transportation mechanisms acceptable to various health facilities and providers, such as commercial courier services and local volunteers whose incidental costs were reimbursed at a standard rate. A rapid reporting mechanism was established to ensure that all test results were promptly communicated back to providers utilizing e-mail and short messaging service (SMS).

Health care providers (both public and private) in the project cities were identified through detailed mapping. The providers were encouraged to prescribe upfront free-of-charge Xpert testing for presumptive infant TB cases that presented to their health facilities. Several sensitization workshops and other advocacy interventions were undertaken with various health providers to increase the project uptake. Providers referred different types of patient specimens (sputum and non- sputum specimen) for Xpert testing using rapid specimen transportation. All presumptive pediatric TB cases with a reported age of less than 2 years that were referred for Xpert testing between April 2014 and October 2016 were included in this analysis. Smear microscopy by Ziehl Neelsen (ZN) staining was done as per routine diagnosis. Xpert testing was done for all types of specimens such as gastric aspirate/lavage (GA/GL), Broncho-alveolar lavage (BAL), cerebrospinal fluid (CSF), induced sputum, lymph node aspirates, pleural fluid, and others. Under this project, case definitions recommended by the Indian National TB Programme were considered.

A presumptive infant TB case was defined as individuals less than two years old with a persistent fever of more than two weeks and weight loss/no weight gain/failure to thrive or presenting with symptoms consistent with pulmonary and/or extra-pulmonary TB.

Bacteriologically confirmed TB cases (TB cases) were defined as having at least one pulmonary and/or extra pulmonary specimen positive for TB by smear microscopy, culture or Xpert MTB/RIF, or other WHO-approved rapid diagnostic test.

Rifampicin-resistant TB cases were defined as bacteriologically confirmed TB cases with indication of rifampicin resistance on one or more of the following assays: Xpert MTB/RIF, line probe assay (LPA) or phenotypic drug susceptibility testing (DST).

For the purpose of analysis, presumptive TB cases with only pulmonary specimens positive for TB were classified as pulmonary TB cases and cases with only extra-pulmonary specimens positive for TB were classified as extra-pulmonary TB cases. Presumptive TB cases with both pulmonary and extra-pulmonary specimens positive for TB were classified as mixed TB cases.

Specimens were collected at referral facilities that were equipped to perform different types of specimen collection procedures. The specimens were collected by trained medical personnel at these centers. In cases where specimens were <1ml, preference was given to Xpert testing ahead of smear microscopy in line with WHO recommendations [20, 21]. For a given patient, whenever multiple types of specimens were available, all types of available specimen were tested. In case of an ‘error’ and/or ‘no result’ test result on Xpert, a repeat test was performed on the remaining sample–buffer mix. In case of ‘invalid’ and ‘rifampicin resistance indeterminate’ test result, repeat testing was performed on a second specimen as per the WHO recommendation [21]. Sputum specimens were tested by adding buffer in 1:2 proportions as recommended by the manufacturer (Cepheid manufacturer instruction). For non-sputum specimens, standard operating procedures (SOPs) developed by the RNTCP and WHO were adopted [22–24, 26]. Confirmatory DST for rifampicin-resistant cases diagnosed on Xpert was performed using line probe assay, Xpert and/or culture DST under the project. Confirmatory DST was performed either on the remnant specimen, and/or additional specimen, if available. All the diagnosed TB cases and rifampicin-resistant TB cases were initiated on appropriate anti-TB regimens.

Feasibility of Xpert implementation was assessed in terms of the ability of the assay to produce a valid result. The absence of a valid test result for any given assay performed was defined as a ‘test failure’ regardless of the underlying reason. The operational feasibility of offering Xpert testing to presumptive infant TB cases through a single lab in each of the four cities was assessed by analyzing the turnaround time (TAT) for specimen transportation, diagnosis and reporting of results to the providers.

### Data management

Data for all presumptive TB cases were collected from routine RNTCP laboratory request forms (S2 File). The project was carried out under programmatic field conditions covering health facilities in the selected geographic area. Data were analyzed using Microsoft Excel 2013 and Epi- Data Analysis (Version 2.1) using R. All confidence intervals were calculated based on the binomial distribution with 95% confidence interval.

### Ethical issues

Xpert testing for presumptive infant TB cases is an approved intervention under RNTCP. The current project was undertaken by FIND, after approval from and in collaboration with RNTCP. As such, the results presented here are our experience-sharing of implementing approved interventions in a programmatic setting within the existing accredited RNTCP TB diagnostic lab network. Since the observations described here are a part of implementation of approved interventions under RNTCP and a part of Standard of TB care in India, separate ethical clearance was not required.

### Funding source

The project was funded by United States Agency for International Development (USAID) under Challenge TB project. FIND was responsible for implementation, training, coordination, monitoring, data analysis and writing of the report in close coordination with Central TB Division, Ministry of Health and Family Welfare, Government of India.

## Results

A total of 7,994 presumptive infant TB cases were tested as part of the project. Of these, 845 (10.6% CI 9.9–11.3) of the subjects were referred from private sector facilities and 76 (1.0% CI 0.8–1.2) had prior history of TB treatment. Samples of 553 (6.9% CI: 6.4–7.5) infants could not be tested on smear microscopy due to limited specimen quantity.

Among the 7,994 enrolled presumptive infant TB cases, a total of 465 (5.8% CI: 5.3–6.4) TB cases were detected. Of the 465 infant TB cases detected, 462 (99.4% CI: 98.1–99.8) were TB positive on Xpert, of which 89 (19.3% CI 15.9–23.1) were also positive on smear microscopy. A total of 3/465 (0.6% CI 0.2–1.9) TB cases were positive on smear microscopy and TB negative on Xpert; since only a single sample of these 3 cases was available, further assessment for the cause of this discordance could not be undertaken. Overall, 25/465 (5.4% CI 3.7–7.8) TB cases had a history of prior TB treatment. TB detection rates were significantly higher in infants enrolled from private sector as compared to public sector (66/845 (7.8% CI 6.2–9.8) vs. 399/7149 (5.6% CI 5.1–6.1)). TB detection rates were similar in male and females (see Table 1).

**Table 1:** Presumptive infant TB cases enrolled under the project and TB cases diagnosed on Xpert, stratified by gender, referring sector and prior history of TB treatment.

Variables		Number of presumptive TB cases tested	Number of TB cases detected TB	Positivity rate %, (95% CI)	No of DR cases	Positivity rate %, (95% CI)
Total		7994	465*	5.8 (5.3, 6.4)	26	5.6 (3.8,8.1)
Gender	Male	4853	255	5.3 (4.7, 5.9)	14	5.5 (3.3,9.0)
	Female	3141	210	6.7 (5.9,7.6)	12	5.7 (3.,9.7)
Sector	Public	7149	399	5.6 (5.1,6.1)	25	6.3 (4.3,9.1)
	Private	845	66	7.8 (6.2,9.8)	1	1.5 (0.3,8.1)
Smear Status	Positive	92	92	100 (95.0, 100)	6	6.5 (3.0, 13.5)
	Negative	7349	344	4.7 (4.2, 5.2)	18	5.2 (3.3, 8.1)
	Not done	553	29	5.2 (3.7, 7.4)	2	6.9 (1.9, 22.0)
Past History of TB Treatment	Yes	76	25	32.9 (23.4, 44.1)	3	12.0 (4.2, 30.0)
	No	7295	401	5.5 (5.0.0, 6.0)	21	5.2 (3.5, 7.9)
	Not available	623	39	6.3 (4.6, 8.4)	2	5.1 (1.4, 16.9)

* Includes 3 smear positive TB results, which were negative on Xpert

Overall, 26/465 (5.6% CI 3.8–8.1) of the infant TB cases detected under the project were found to be rifampicin-resistant TB cases (RR-TB). Of the detected RR-TB cases, the majority did not have any prior history of TB treatment, and were negative on smear microscopy. RR-TB detection rates were similar in male and female infant TB cases (see Table 1). Confirmatory DST for RR-TB cases diagnosed on Xpert could be performed for 23 cases (3 lost-to-follow-up) using line probe assay and/or culture DST under the project (Table A in S1 File) with high levels of concordance on LPA and culture for this with valid results (9/11 [82%] and 2/2 [100%], respectively).

Of the 465 infant TB cases detected under the project, 349 (75.1% CI: 70.9–78.8) were pulmonary TB cases of which 17 (4.9% CI 3.1–7.7) were RR-TB; 93 (20.0% CI 16.6–23.9) TB cases were extra-pulmonary TB cases of which 6 (6.5% CI 3.0–13.4) were RR-TB. A total of 23 (4.9% CI: 3.3–7.3) TB cases had both pulmonary and extra pulmonary specimens positive for TB, of which 3 (13.0% CI:4.5–32.1) were rifampicin-resistant. A total of 40 (8.6% CI: 6.4–11.5) of the 465 TB cases were cases of TB meningitis of which 6 (15.0% CI: 7.1–29.1) were found to be RR-TB.

Further analysis was done to assess Xpert performance in comparison to smear microscopy on different types of specimens. A total of 8881 specimens collected from 7994 infants were tested on Xpert. The majority specimen tested were non-sputum specimens and only 6.1% (CI: 5.6–6.6) were sputum specimens. The sputum samples were either collected by induction or naso-pharyngeal aspiration. Amongst the non-sputum specimens, 71.5% (CI 70.5–72.4) were gastric aspirate/lavage, 12.5% (CI 11.8–13.2) were CSF, and 4.2% (CI 3.8–4.7) were BAL (see Table 2). Valid results were provided for 99.7% of the cases by ensuring retesting of initial test failures (initial failures-209 patients; 2.6% and after retesting 0.25%). Xpert was 4.3-fold more likely to be positive as compared to smear microscopy, independent of sample type (5.7% (CI 5.2–6.2) vs. 1.3%, (CI 1.0–1.5). Overall, lymph nodes/tissue, pus, BAL, GA/GL, CSF and sputum had a high likelihood of being positive. Low TB detection rates were observed on pleural fluid and ascitic fluid.

**Table 2:** Comparison of Xpert and smear positivity in different types of specimen.

Specimens	No. tested on Xpert	Proportion of all Xpert tested (95% CI)	Xpert Positives	Xpert positivity rate % (95% CI)	No. tested with smear	Proportion of all smear tests	Smear Positives	Smear positivity rate % (95%CI)	Rif Resistant	% of Rifampicin resistant %
Gastric aspirate & Gastric lavage	6349	71.5(70.5,72.4)	314	5.0 (4.4, 5.5)	5612	75.5 (74.6,76.5)	56	1 (0.8,1.3)	22	7.0(4.7–10.4)
Sputum & Induced Sputum	539	6.1(5.6–6.6)	23	4.3(2.9,6.3)	451	6.1 (5.6, 6.6)	7	1.6 (0.8, 3.2)	4	17.4(7.0–37.1)
Cerebrospinal fluid (CSF)	1112	12.5 (11.8,13.2)	52	4.7(3.6,6.1)	738	9.9 (9.3, 10.6)	0	0	8	15.4 (8.0–27.5)
Pleural Fluid	160	1.8 (1.5,2.1)	3	1.9 (0.6,5.4)	122	1.6 (1.4, 2.0)	0	0		
Bronco alveolar lavage (BAL)	377	4.2 (3.8,4.7)	48	12.7(9.7, 16.5)	282	3.8 (3.4, 4.3)	11	3.9 (2.2,6.8)	1	2.1(0.4–10.5)
Pus	98	1.1 (0.2,1.3)	31	31.6 (23.3, 41.4)	78	1.1 (0.8, 1.3)	12	15.4 (9.0,25.0)	2	6.5(1.8–20.7)
Lymph Node	34	0.4 (0.3,0.5)	12	35.3 (21.5, 52.1)	22	0.3 (0.2,0.4)	4	18.2 (7.3,38.5)		
Ascitic Fluid	41	0.5 (0.3,0.6)	1	2.4 (0.4, 12.6)	28	0.4 (0.3,0.5)	0	0		
Tissue	23	0.3 (0.2,0.4)	3	13.0 (4.5, 32.1)	17	0.2 (0.1,0.4)	1	5.9 (1.0,27.0)		
Others*	148	1.7 (1.4, 2.0)	20	13.5 (8.9, 20.0)	78	1.1 (0.8, 1.3)	2	2.6 (0.7, 8.9)	0	
**Total**	**8881**		**507**	**5.7 (5.2,6.2)**	**7428**		**93**	**1.3 (1.0,1.5)**	**37**	**7.3 (5.3–9.9**

*Others included pericardial, Endotracheal tube extraction, Tracheal aspirate and urine specimens.

**Fig 1:**
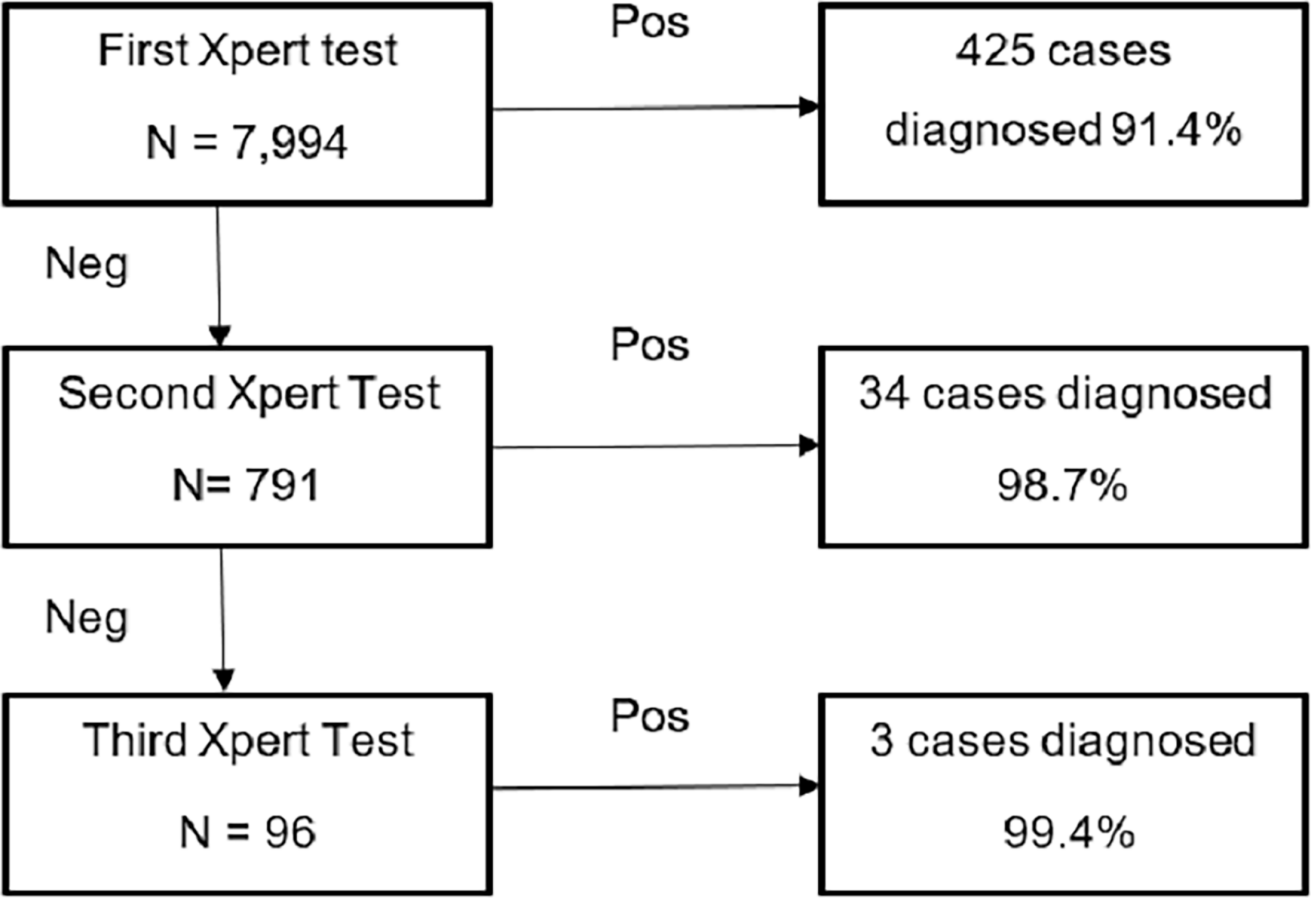
Incremental case detection by testing multiple specimen.

It was further observed that testing multiple specimens per patient on Xpert led to incremental TB case detection. Of the 7,994 infants, 7203 patients had a single specimen tested, for 695 patients two specimens were tested, and for 96 patients three specimens were tested. Overall, 462 infant TB cases were detected under the project on Xpert. All 7,994 patients received a first Xpert test. In total, 791 and 96 patients gave a second and third specimen for testing, respectively. Of the 465 patients, 425 (91.4%) were detected on the first Xpert test, and an additional 34 cases after the second Xpert test, with a cumulative detection of 98.7% cases and an incremental yield of 7.3% on a second Xpert test. An additional 3 cases were diagnosed after the third test, i.e. incremental yield of 0.7% (Fig 1).

All the collected specimens were transported to the central testing facility in each of the 4 cities on the day of collection; all received specimens were tested on Xpert on the day of receipt at the laboratory and test results were reported on day of testing. The median time between reporting of test results and treatment initiation was observed to be 2 days for both rifampicin-sensitive (IQR: 1–4) and rifampicin-resistant (IQR: 2–10) TB case (see Table 3).

**Table 3:** Project turnaround time: Median time between events in diagnostic cascade, Median (IQR).

Variables		Days between collection and receipt	Days between receipt and testing	Days between testing and reporting	Days between reporting and treatment initiation (for those who initiated treatment after reporting)	Days between reporting and treatment initiation for DR TB (for those who initiated treatment after reporting)
Total		0 (0,0)	0 (0,0)	0 (0,0)	2 (1,4)	2 (2,10)
Gender	Male	0 (0,0)	0 (0,0)	0 (0,0)	2 (1,4)	2 (2,7)
	Female	0 (0,0)	0 (0,0)	0 (0,0)	2 (1,4)	2 (1,7)
Sector	Public	0 (0,0)	0 (0,0)	0 (0,0)	2 (1,5)	2 (2,9)
	Private	0 (0,0)	0 (0,0)	0 (0,0)	1 (1,2)	10 (10,10)

92.4% of specimens were transported on the same day

95.7% of specimens were tested within 1 day of receipt

93.4% of specimens were reported on the same day as testing

92.5% of patients were initiated on treatment within 11 days

90.0% of MDR patients were initiated on treatment within 15 days

Overall, 439 of 465 infant TB cases diagnosed (94.4% CI 91.9–96.2) were rifampicin-sensitive TB cases. Of these, 363 (82.7%) TB cases were put on first-line anti-TB treatment (ATT) (see Table 4). Before the start of treatment amongst the diagnosed rifampicin-sensitive TB cases, 22 (5.0% CI: 3.3–7.5) infants had died and 22 (5.0% CI: 3.3–7.5) were lost to follow-up before treatment initiation. Three cases (0.7%, CI 0.2–2.0) were ‘transferred out’ for treatment initiation to their respective TB project management units under RNTCP. A total of 29 (6.6% CI: 4.6–9.3) infants could not be traced for confirmation of treatment initiation. Similarly, of the total 26 rifampicin-resistant TB cases, 22 (84.6% CI 66.5–93.8) were put on treatment for multidrug-resistant tuberculosis (MDR-TB) (see Table 4). three infants died before the start of treatment and one was lost to follow-up prior to treatment initiation.

**Table 4:** Treatment initiation status of bacteriologically confirmed TB cases.

Rifampicin sensitive TB cases											
Variables	Grand Total	On Treatment	Proportion on treatment,% (95% CI)	Number Died	Proportion died,% (95% CI)	Lost to follow-up	Proportion Lost to follow-up,% (95% CI)	Not Traceable	Proportion Not traceable,% (95% CI)	Referred Out	Proportion referred out,% (95% CI)
Male	241	206	85.5 (80.5–89.4)	8	3.3 (1.7–6.4)	8	3.3 (1.7, 6.4)	16	6.6 (4.1–10.5)	3	1.2 (0.4,3.6)
Female	198	157	79.3(73.1–84.4)	14	7.1 (4.3–11.5)	14	7.1 (4.3, 11.5)	13	6.6 (3.9–10.9)	0	0 (0, 19)
Private	65	59	90.8 (81.3–95.7)	0	0 (0, 5.6)	2	3.1 (0.8, 10.5)	4	6.2 (2.4–14.8)	0	0 (0,5.6)
Public	374	304	81.3(77.0–84.9)	22	5.9 (3.9–8.7)	20	5.3 (3.5, 8.1)	25	6.7 (4.6–9.7)	3	0.8 (0.3, 2.3)
Total	439	363	82.7 (78.9–85.9)	22	5.0 (3.3–7.5)	22	5 (3.3, 7.5)	29	6.6 (4.6–9.3)	3	0.7 (0.2, 2.0)
Rifampicin resistant TB cases											
Male	14	12	85.7(60.1–96.0)	1	14.3 (4.0,39.9)	1	7.1 (1.3–31.5)				
Female	12	10	83.3 (55.2–95.3)	2	16.7 (4.7–44.8)	0	0				
Private	25	21	84.0 (65.3–93.6)	3	12 (4.2–30.0)	1	4 (0.7–19.5)				
Public	1	1	100 (5.5,100)	0		0	0				
Total	26	22	84.66.5–93.8)	3	11.5 (4.0–29.0)	1	3.8 (0.7,18.9)				

A total 51 (11.0% CI 8.4–14.1) of the 465 infant TB cases had died by the time of data closure for analysis (Table B in S1 File). Of these 25/51(49% CI: 35.9–62.3) TB cases had died before the start of treatment and another 26/51 (51% CI: 37.7–64.1) had died after starting treatment. Of 51 cases of infant mortality, 6 were rifampicin resistant. Exact date of death could be verified for 9 patients. Overall, 29 of the 42 deaths (69.0% CI: 54.0–80.9) occurred within two weeks of specimen collection.

Of the notified TB cases initiated on treatment, the majority were not eligible for outcome as they had not completed 6 months of treatment at the time of analysis. Similarly, of the 22 RR-TB cases that were initiated on treatment, 14/22 (63.6% CI 43.0–80.3) were still on treatment and not eligible for treatment outcome. Since the majority of cases were not eligible for treatment outcome, this aspect was excluded from the scope of current manuscript.

## Discussion

A key bottleneck in prompt TB diagnosis in infants is related to practical challenges in obtaining a sputum specimen, delay in recognizing symptoms as of TB and the need for collection of alternative types of specimen in these young children [27, 28]. The current project was implemented under programmatic conditions, covering a large number of facilities across four major cities of India, with different types of specimens from presumptive infant TB cases routinely collected and subjected to upfront Xpert testing under the project. Greater than 90% of specimens were non-sputum specimens. Xpert assay provided a high proportion of valid results on different types of specimens, with 5.8% TB detection rate.

These findings are similar to other studies conducted using Xpert assay on sputum and non-sputum specimens collected from presumptive pediatric TB cases [29–33]. The proportion of valid interpretable results on Xpert under the current study was higher than previous reports from India and Germany [31, 34]. These results demonstrate the feasibility of collecting different types of specimen from infants and utility of upfront testing on Xpert as compared to other available diagnostic tests. Our findings are in line with the recommendations from a meta-analysis by Detjen et al [19] in pediatric TB cases, which advocated for the scale-up of Xpert in order to improve access to rapid diagnosis for children. Similar to other studies, higher positivity rates were observed in specimens such as gastric aspirate/gastric lavage, CSF, fine needle aspirates of lymph nodes, Pus, BAL and tissue specimens in India [35] and other countries [34, 36–41] in pediatric TB cases. The positivity rate on ascitic fluid and pleural fluid observed in our study was very low which is consistent with guidance issued by WHO [24] and observations in other studies [42, 43], suggesting limited utility in extending Xpert testing to these specimens. The higher percentage of detection that was observed on BAL samples compared to other pulmonary samples might be due to children with more severe disease being subjected to bronchoscopy/BAL. We also observed that a higher proportion of GA/GL samples were collected in the public sector as compared to private sector (75% vs 44%). Similarly, BAL and sputum/IS samples collected in private sector were greater than the public sector (29% vs 8%).

Under the study, incremental TB case detection by testing multiple patient specimens was observed. We observed that with testing a second patient specimen there was a significant increase in TB detection rates, while testing a third specimen provided limited additional gain in TB detection.

Upfront Xpert testing for presumptive infant TB cases led to detection of significant numbers of TB and DR-TB cases. Other studies implemented in the pediatric age group have documented similar utility of Xpert in detecting TB [25, 36, 37]. In the current project, lower TB and DR-TB detection rates as compared to our earlier publications in the pediatric population (0–14 yrs) could be attributed to greater degree of non-specificity in the clinical symptoms associated with this age-group [25].

There are very limited reports of DR-TB in the infant population. Under the large project cohort of infants, high levels of rifampicin-resistance in TB cases were observed; most of the DR-TB cases being treatment naïve, suggesting primary transmission of DR-TB. The levels of DR-TB were high across the four cities, and didn’t show any clustering around particular facilities or period of time or sector (public or private). However, the project did not draw a representative sample of the infant population suffering with TB and the observed levels are not representative of the levels of DR-TB in infants in India or the project cities. However, high levels of DR-TB in young children flags the need to further research on this aspect, and the need to provide evidence-based diagnosis to this highly vulnerable population in contrast to the current prevailing practice where the majority of TB cases are treated empirically.

The project also demonstrated the ability to achieve rapid turn-around times (TAT) through a hub and spoke model. All specimens were transported, tested and results reported on the day of specimen collection, while catering to presumptive infant TB cases from various facilities across four large cities of India. This rapid TAT was achieved by developing locally the most feasible rapid transportation mechanisms, utilizing services of volunteers, having high throughput labs, having them functional for extended hours as and when required, and electronic reporting of results. This in turn facilitated prompt treatment initiation. These findings demonstrate the feasibility and need for replicating a similar service delivery model in other similar settings.

In the current analysis, we observed that 82.8% of the notified infant TB cases were initiated on treatment. However, in spite of the same day diagnostic TAT and prompt initiation of treatment, 11.0% of the infant TB cases had died by the time of project data closure. Majority (~75%) of these infant deaths happened within 15 days of prescription of Xpert testing which is a major cause of concern, suggesting delays in the pediatric patient pathways that must be shortened for better infant TB diagnosis and case management. High mortality rates observed here are similar to reports from Sub-Saharan African region [44–46]. Further improvements in achieving early diagnosis will be necessary for countries to achieve the global goals for the control of childhood TB [47].

### Limitations

The study enrolled only those infants whose samples were referred by the providers engaged under the project. Therefore, the study finding might not be a representative sample of infants in India. Though this limitation is important, there is limited available data about TB diagnosis using Xpert among infants and the large sample size enrolled in different settings under this project provides useful insights. Furthermore, this study was a pragmatic study and infants were not diagnosed in a standardized way, which needs to be taken into account when considering the yield of diagnosis and the incremental value of additional testing. Further, for Xpert negative patients, the treating provider was given the option for sending additional samples for testing. However, if all testing remained negative, it was up to the provider to prescribe additional tests and/or empirical treatment. As a part of the upfront sensitization/training package for the referring providers, all providers were well familiarized with WHO guidance on Xpert, including sensitivity limitations of Xpert in that a negative result does not rule out TB. Access to quality TB treatment was ascertained for positive cases on GeneXpert under the project.

Reconfirmation of RIF resistant cases was attempted for most of the cases; however, valid results could be obtained only for 13 of the 23 specimens due to non-availability of additional specimens. In the specimens with valid results we observed high level of concordance, however due to small sample size and large proportion of specimens on which reconfirmation could not be done, we are challenged to draw any statistical conclusion.

## Conclusion

This study demonstrated that Xpert can be a promising tool for infant TB and DR-TB detection. The project highlighted the feasibility of collecting non-sputum specimens and rolling out rapid and upfront Xpert testing for presumptive infant TB cases. The hub and spoke model ensured a rapid TAT for results, which in turn facilitated prompt and appropriate treatment initiation. The study highlights two major causes for concern that need to be further addressed: the high levels of rifampicin-resistance and the high proportion of early treatment mortality in infant TB cases. The former underscores the need for scaling-up upfront Xpert access to presumptive infant TB cases and monitoring of infant RR-TB case from epidemiological perspective. The latter highlights the need for systematically studying infant TB care pathways, for overall improvement in earlier presentation to services and high-quality TB care for this vulnerable population.

## Supporting information

S1 File Supplementary information. Table A: Confirmatory DST for RR-TB cases diagnosed on Xpert under the project
Table B: Mortality Data (Mortality status of notified TB and DR TB cases).
(DOCX)

S2 File Annex RNTCP. (PDF)
