# Supplementary material for: Upfront Xpert MTB/RIF testing on various specimen types for presumptive infant TB cases for early and appropriate treatment initiation
Source: PLoS One. 2018 Aug 30;13(8):e0202085. doi: 10.1371/journal.pone.0202085 (PMC6116934; doi:10.1371/journal.pone.0202085)
Supplement: S1 File — Table A: Confirmatory DST for RR-TB cases diagnosed on Xpert under the project Table B: Mortality Data (Mortality status of notified TB and DR TB cases). (DOCX) [file pone.0202085.s001.docx]

# **S1 Supporting information**

Table A: Confirmatory DST for RR-TB cases diagnosed on Xpert under the project

| **Patient ID** | **LPA** | **Culture** |
| --- | --- | --- |
| 1 | Concordant | Not Done |
| 2 | Negative result | Not Done |
| 3 | Concordant | Not Done |
| 4 | Negative result | Contaminated |
| 5 | Negative result | Negative result |
| 6 | Not Done | Negative result |
| 7 | Not Done | Concordant |
| 8 | Concordant | Not Done |
| 9 | Concordant | Negative result |
| 10 | Negative result | Contaminated |
| 11 | Concordant | Negative result |
| 12 | Discordant | Negative result |
| 13 | Not Done | Concordant |
| 14 | Concordant | Negative result |
| 15 | Negative result | Negative result |
| 16 | Negative result | Not Done |
| 17 | Concordant | Negative result |
| 18 | Concordant | Negative result |
| 19 | Concordant | Not Done |
| 20 | Discordant | Negative result |
| 21 | Negative result | Negative result |
| 22 | Negative result | Not Done |
| 23 | Invalid | Not Done |

Of the 26 DR-TB patients, 23 were subjected reconfirmation on at least one of the tests- culture/LPA. LPA was concordant in 9 of 11 specimens with valid results & Culture on 2 of 2 specimens with valid results.

Ct values were not available for the RIF-resistant specimens.

**Concordance by method -only for valid results**

| **% concordant by LPA** | **81.8%** |
| --- | --- |
| **% concordant by culture** | **100.0%** |

Table B: Mortality Data (Mortality status of notified TB and DR TB cases)

| **Variables** | **Pre Treatment mortality** | | | **Post Treatment mortality** | | |
| --- | --- | --- | --- | --- | --- | --- |
|  | **Rif Sen** | **Rif Res** | **Total** | **Rif Sen** | **Rif Res** | **Total** |
| Sex |  |  |  |  |  |  |
| Female | 14 | 2 | 16 | 11 | 3 | 14 |
| Male | 8 | 1 | 9 | 12 | 0 | 12 |
| Past history of treatment |  |  |  |  |  |  |
| No | 21 | 3 | 24 | 21 | 2 | 23 |
| Yes | 1 | 0 | 1 | 2 | 1 | 3 |
| Total | 22 | 3 | 25 | 23 | 3 | 26 |
