# Supplementary material for: Upfront Xpert MTB/RIF testing on various specimen types for presumptive infant TB cases for early and appropriate treatment initiation
Source: PLoS One. 2018 Aug 30;13(8):e0202085. doi: 10.1371/journal.pone.0202085 (PMC6116934; doi:10.1371/journal.pone.0202085)
Supplement: S2 File — (PDF) [file pone.0202085.s002.PDF]

RNTCP Request Form for examination of biological specimen for TB

(Required for Diagnosis of TB, Drug Sensitivity Testing and follow up)

**Patient Information**			
**Patient name**		**Age (in yrs):____**	**Gender: ☐M ☐F ☐TG**
**Patient mobile no. or other contact no.**		**Specimen date of collection** (DD/MM/YY) ________	**☐ Sputum** **☐Other (specify)______**
**Aadhar no.**			
**Patient address with landmark**		**HIV Status: ☐Reactive ☐Non-Reactive ☐Unknown**	
		**Key populations: ☐Contact of known TB Patient ☐Diabetes ☐Tobacco ☐Prison ☐Miner ☐Migrant ☐Refugee ☐ Urban slum ☐Health-care worker ☐Other(specify) ______**	

**Name and Type of referring facility** (PHI/DMC/TU/ DTC/ICTC/ART/Medical College/DR-TB Centre/Private Others, specify): _______________________ **Health Establishment ID (NIKSHAY): _ _ _ _**	**CDL NIKSHAY ID: _ _ - _ _ _ - _ - C -_ _- _ _ _ _ _** **RNTCP TB Reg No. ___________________** **Or** ☐ Not Applicable
**State: ______________________ District:_______________ Tuberculosis Unit (TU): _________________**	

Reason for Testing:

**Diagnosis and follow up of TB**		
Diagnosis (NIKSHAY ID__________________)		Follow up (Smear and culture)
H/O anti TB Rx for >1 month: ☐ Yes ☐ No		RNTCP TB Reg No _______________ NIKSHAY ID:_________________
☐ Presumptive TB ☐ Repeat Exam ☐ Private referral ☐ Presumptive NTM	Predominant symptom ____________________ Duration ______ days	Regimen: ☐New ☐ Previously Treated Reason: ☐ End IP ☐ End CP Post treatment: ☐ 6m ☐ 12m ☐ 18m ☐ 24m

**Diagnosis and follow up Drug-resistant TB**		
Drug Susceptibility Testing (DST)		Follow up (Culture)
Presumptive MDR TB	☐ New ☐ Previously treated<br>☐ At diagnosis<br>☐ Contact of MDR/RR TB<br>☐ Follow up Sm +ve<br>☐ Private referral<br>☐ Discordance resolution	PMDT TB No _____________<br>DR TB NIKSHAY ID: ________________<br>Regimen:<br>☐ Regimen for INH mono/poly resistant TB<br>☐ Regimen for MDR/RR TB<br>☐ Shorter regimen*<br>☐ Modified Regimen for MDR/RR-TB + FQ /SLI resistance<br>☐ Regimen for XDR TB<br>☐ Modified Regimen for mixed pattern resistance<br>☐ Regimen with New Drug for MDR-TB Regimen + FQ/SLI resistance<br>☐ Regimen with New Drug for XDR-TB<br>☐ Regimen with New Drug for failures of regimen for MDR TB<br>☐ Regimen with New Drug for failures of regimen for XDR-TB<br>☐ Regimen with New Drug for mixed pattern resistance<br>Treatment ☐ Month ☐ Week :_____________
☐ Presumptive H mono/poly		
Presumptive XDR TB	☐ MDR/RR TB at Diagnosis<br>☐ ≥ 4 months culture positive<br>☐ 3 monthly for persistent culture positives (treatment month _____)<br>☐ Culture reversion<br>☐ Failure of MDR/RR-TB regimen<br>☐ Recurrent case of second line treatment<br>☐ Discordance resolution	

Test requested:

☐Microscopy ☐TST ☐IGRA ☐Chest X-ray ☐Cytopathology ☐Histopathology ☐ CBNAAT ☐ Culture ☐DST ☐Line Probe Assay ☐Gene Sequencing ☐Other (Please Specify) ______________________

Requestor Name, Designation and Signature: ____

Contact Number:_________________ Email ID:_______________________

Results: **NIKSHAY ID** Generated: _______________________ **CDL NIKSHAY ID:** _______________

**Microscopy** (☐ ZN ☐ Florescent)							
	Lab Sr. No	Visual appearance	Result				
			Negative	Scanty	1+	2+	3+
Sample A							
Sample B							

Date tested: ______________ **Date Reported:**_______________ **Reported by:**______________________

(Name and Signature)

Cartridge Based Nucleic Acid Amplification Test (CBNAAT)

**Sample**	☐ A	☐ B		
**M. Tuberculosis**	☐ Detected	☐ Not Detected	☐ N/A	
**Rif Resistance**	☐ Detected	☐ Not Detected	☐ Indeterminate	☐ N/A
**Test**	☐ No Result	☐ Invalid	☐ Error – Error Code________	(Please arrange for fresh sample)

Date tested: ______________ **Date Reported:**_______________ **Reported by:**_______________________

(Name and Signature)

Culture (☐ LJ ☐ LC)

Lab Sr. No	Negative	Positive	NTM (write species)	Contamination

Date Result: ______________ **Date Reported:**_______________ **Reported by:**_______________________

(Name and Signature)

Line Probe Assay (LPA)

☐ Direct ☐ Indirect Lab serial __________

First line LPA

RpoB: ---- **locus control:** ☐ present ☐ absent

WT1: ☐present ☐ absent **WT2:** ☐ present ☐ absent **WT3:** ☐ present ☐absent **WT4:** ☐ present ☐ absent

WT5: ☐ present ☐ absent **WT6:** ☐ present ☐absent **WT7 :** ☐ present ☐absent **WT8 :** ☐ present ☐ absent

MUT1 (D516V): ☐ present ☐absent **MUT2A (H526Y):** ☐ present ☐absent **MUT2B (H526D):** ☐ present ☐absent **MUT3 (S531L):** ☐ present ☐absent

Kat G: ----- locus control: ☐ present ☐ absent

WT1 (315): ☐ present ☐ absent

MUT1 (S315T1): ☐ present ☐ absent
MUT2 (S315T2): ☐ present ☐ absent

Inh A:----- locus control: ☐present ☐absent

WT1 (-15, -16): ☐ present ☐ absent **WT2 (-8):** ☐ present ☐ absent

MUT1 (C15T): ☐ present ☐ absent **MUT2 (A16G):** ☐ present ☐ absent
MUT3A (T8C): ☐ present ☐ absent **MUT3B (T8A):** ☐ present ☐ absent

Second line LPA

gyrA:--

locus control: ☐ **present** ☐ **absent**

WT1 (85-90): ☐ **present** ☐ **absent**
WT2 (89-93): ☐ **present** ☐ **absent**
WT3 (92-97): ☐ **present** ☐ **absent**

MUT1 (A90V): ☐ **present** ☐ **absent**
MUT2 (S91P): ☐ **present** ☐ **absent**
MUT3A (D94A): ☐ **present** ☐ **absent**
MUT3B (D94N/Y): ☐ **present** ☐ **absent**
MUT3C (D94G): ☐ **present** ☐ **absent**
MUT3D (D94H): ☐ **present** ☐ **absent**

gyrB:----

locus control: ☐**present** ☐**absent**

WT1 (536-541): ☐ **present** ☐ **absent**

MUT1 (N538D): ☐ **present** ☐ **absent**
MUT2 (E540V): ☐ **present** ☐ **absent**

rrs:-----

locus control: ☐**present** ☐**absent**

WT1 (1401-02): ☐ **present** ☐ **absent**
WT2 (1484): ☐ **present** ☐ **absent**

MUT1 (A1401G): ☐ **present** ☐ **absent**
MUT2 (G1484T): ☐ **present** ☐ **absent**

eis:-----

locus control: ☐**present** ☐**absent**

WT1 (37): ☐ **present** ☐ **absent**
WT2 (14, 12, 10): ☐ **present** ☐ **absent**
WT3 (2): ☐ **present** ☐ **absent**

MUT1 (C-14T): ☐ **present** ☐ **absent**

Final LPA Interpretation: ---

MTB result ☐ MTB positive ☐ MTB Negative
RIF ☐ Sensitive ☐ Resistant ☐ Indeterminate **INH** ☐Sensitive ☐Resistant ☐ Indeterminate
Quinolone ☐ Sensitive ☐ Resistant ☐ Indeterminate **SLID** ☐Sensitive ☐Resistant ☐ Indeterminate

Date Result: ______________ **Date Reported:**_______________ **Reported by:**_______________________

(Name and Signature)

Drug Susceptibility Test (DST) results

Lab Sr.No	1st line drugs						SLI			FQ			Other										
	R	H (inhA)	H (katG)	S	E	Z	Km	Cm	Am	Lfx	Mfx (0.5)	Mfx (2)	Eto	PAS	Lzd	Cfz	Clr	Azi					

Date Result: ______________ **Date Reported:**_______________ **Reported by:**_______________________

R: Resistant; S: Susceptible; C: Contaminated; **--** *Not done* **(Name and Signature)**

Other tests for TB diagnosis

Test (Please Specify): ______________________
Result:____

____

Date reported:_______________ **Reported by:**_______________________

(Name and Signature)
